# Effects of ingesting Dyma-Burn Xtreme, a thermogenic dietary supplement on metabolic rate and subjective measures of mood state

**DOI:** 10.1186/1550-2783-9-S1-P31

**Published:** 2012-11-19

**Authors:** Stacie Urbina, Craig Jones, Sara Hayward, Cliffa Foster, Shawn Wells, Rob Wildman, Bill Campbell, Lem Taylor, Colin Wilborn

**Affiliations:** 1 University of Mary Hardin-Baylor, TX, 76513, USA; 2 Dymatize Nutrition, Dallas, TX, 75234, USA; 3 University of South Florida, Tampa, FL, 33544, USA

## Background

Many supplements on the market today contain ingredients that claim to increase metabolism and enhance fat loss. Green tea extract and caffeine have well known thermogenic properties. The purpose of this study was to evaluate the effects of proprietary thermogenic dietary supplement Dyma-Burn Xtreme, containing a blend of ingredients including caffeine, green tea extract, raspberry ketones and L-carnitine, on resting energy expenditure and subjective measures of alertness, focus, energy, fatigue, concentration, and hunger.

## Methods

In a double-blind, crossover design 6 male and 6 female subjects (N = 12, 22 ± 9.5 yrs, 171 ± 11.2 cm, 76.9 ± 11.2 kg, 22.7 ± 9.5), consumed either a 2 capsule serving of Dyma-Burn Xtreme (DBX) or placebo (PLC). Subjects arrived at the lab on a 12 hour fast at 8:00am and had a baseline resting energy expenditure (REE), respiratory exchange ratio (RER), and mood state questionnaire assessed. Subjects then consumed either DBX or PLC and REE and RER were assessed in a supine position for 25 minutes, and questionnaire were assessed at 1-hour (1HR), 2-hours (2HR), 3-hours (3HR), and 4-hours (4HR) post consumption. All data was analyzed utilizing a 2X5 ANOVA and one-way ANOVA’s were used in the case of a significant interaction. A Kruskal Wallis one-way analysis of variance was used for all survey data. A significance value of 0.05 was adopted throughout.

## Results

A significant time effect and group x time interaction effect were observed among groups for changes in REE (p > 0.05). Post-hoc analyses revealed REE levels were significantly different at the 1HR (DBX: 123.4 ± 78.2 vs. PLC: -3.1 ± 88.4 kcal/day), 2HR (DBX: 125.5 ± 62.2 vs. PLC: -20.3 ± 72.6 kcal/day), 3HR (DBX: 142.4 ± 101.1.6 vs. PLC: 9 ± 114.77 kcal/day), and 4HR (DBX: 147.3 ± 83.5 vs. PLC: 32.1 ± 86.7 kcal/day) indicating a more profound metabolic response from DBX. There was no significant (p < 0.05) time or interaction effect for RER. Questionnaire data revealed significant increases in alertness and focus (p< 0.05) at the 1HR time point, energy at the 1HR & 2HR time points, and decreases in fatigue at the 1HR time point. There were no significant changes in hunger or concentrations.

## Conclusions

The results of this study revealed that the proprietary blend Dyma-Burn Xtreme® is capable of increasing energy expenditure over a four hour period. In addition, markers of mood state such as focus, alertness, and energy showed significant improvements over a two hour period.

